# Development and validation of postoperative and preoperative platelets ratio (PPR) to predict the prognosis of patients undergoing surgery for colorectal cancer: A dual‐center retrospective cohort study

**DOI:** 10.1002/cam4.4930

**Published:** 2022-06-11

**Authors:** Wei Yang, Xiaoying Zheng, Minghui Wu, Fengming Zhang, Shuizhi Xu, Xiuchao Wang, Menghui Song, Chang You, Ting Zhang, Minghua Jiang, Chunming Ding

**Affiliations:** ^1^ School of Laboratory Medicine and Life Science Wenzhou Medical University Wenzhou Zhejiang China; ^2^ Key Laboratory of Laboratory Medicine, Ministry of Education Wenzhou Medical University Wenzhou Zhejiang China; ^3^ Clinical Laboratory Center Taizhou First People's Hospital Taizhou Zhejiang China; ^4^ Clinical Laboratory Center The Second Affiliated Hospital & Yuying Children's Hospital of Wenzhou Medical University Wenzhou Zhejiang China

**Keywords:** colorectal cancer, postoperative and preoperative platelets ratio, preoperative platelet count, prognosis, validation

## Abstract

**Background:**

Platelets occupy a prominent place in tumor proliferation and metastasis, and platelet count is relevant to the prognosis of tumor patients. But preoperative platelet counts cannot be standardized and individualized due to the variability among individuals, instruments, and regions, and the connection between postoperative platelet count and prognosis remains unknown. A standardized indicator of platelet count was designed to forecast the prognosis of colorectal cancer (CRC).

**Methods:**

Five hundred and eighty six patients who suffered radical resection of CRC between 2013 and 2019 were collected. A development‐validation cohort of standardized and individualized platelet counts for prognostic assessment of CRC was designed. We first determined the ability of PPR and other peripheral blood count‐related indicators to predict the mortality of patients with CRC and validated them in a separate cohort. Kaplan–Meier analysis was executed to evaluate the survival and univariate and multivariate analyses were executed to explore the relevance. Time‐dependent ROC was measured to estimate the predictive usefulness. Decision curve analysis was used to verify the clinical net benefit.

**Results:**

Important baseline variables showed a similar distribution in two independent queues. In the development cohort, postoperative platelet count and postoperative/preoperative platelets ratio (PPR) were independent predictors of prognosis in CRC patients. PPR showed the largest area under the curve (AUC) in evaluating 1‐year and 5‐year OS (AUC: 0.702 and 0.620) compared to others. In the validation cohort, platelet/lymphocyte ratio and PPR were validated to be independently concerned about OS of CRC patients and PPR showed the largest AUC in evaluating 1‐year and 3‐year OS (AUC: 0.663 and 0.673). PPR and joint index of platelet count and PPR showed better predictive value and clinical net benefit.

**Conclusions:**

PPR has been identified and validated to be independently concerned about OS of patients with CRC and was a reliable and economic indicator to evaluate the prognosis.

## Graphical abstract

In this dual‐center retrospective study, the authors propose a new predictive model and validate its association with colorectal cancer. Postoperative platelet/preoperative platelet ratio (PPR) is significantly associated with the risk of colorectal cancer and has a good predictive value for prognosis. This study provides additional evidence for the utility of platelet count in colorectal cancer risk stratification.

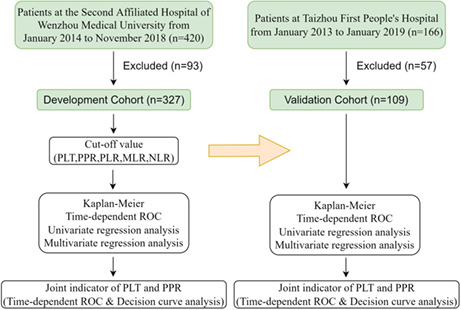

## 1 INTRODUCTION

Colorectal cancer (CRC) is the third most frequent cancer and the second most lethal cancer worldwide.^1^ The tumor, nodes, metastasis (TNM) is still the globally recognized benchmark for cancer classification, prediction, management, and clinical trial and research.^2^ However, significant differences have been observed in clinical outcomes in patients with the same TNM stage,^3^ which demonstrates that more markers are still needed for further tumor stratification and individualized clinical outcome prediction.

Platelets are the second most numerous cells of the peripheral blood and are immensely correlated with cancer cell proliferation, invasiveness, and metastasis.^4^, ^5^, ^6^, ^7^ Recent studies have suggested that preoperative platelet count (PLT) was closely related to survival outcomes for cancer patients, such as CRC,^8^, ^9^, ^10^, ^11^ lung cancer,^12^ and esophageal squamous cell carcinoma.^13^ However, different regions or units have distinct definitions of the normal range of platelet count, such as 100–300 × 10^9^/L,^14^ 125–350 × 10^9^/L,^15^ and 150–400 × 10^9^/L.^16^ In addition, significant differences existed in platelet counts among ethnicity, age, and gender.,^17^ hampering the application of preoperative platelet count as a prognostic marker in clinical practice and individualized prediction. Therefore, we considered whether the postoperative/preoperative platelet ratio (PPR) could be used as a potential indicator for clinical practice and individualized prediction in the prognosis of patients with CRC.

Large quantities of studies have supported that various parameters of peripheral blood cell count are inflammatory parameters to estimate the survival outcomes of patients with cancer. These indicators include the neutrophil/lymphocyte ratio (NLR),^18^, ^19^ platelet/lymphocyte ratio (PLR),^20^, ^21^, ^22^ and monocyte/lymphocyte ratio (MLR).^23^, ^24^ However preoperative parameters alone cannot accurately predict patient survival outcomes because disease progression is a dynamic process. And these indicators cannot be standardized due to differences in detection methods and instruments in different units. Hence, a development‐validation cohort of standardized and individualized platelet counts for prognostic assessment of CRC was designed. And found that it was independently concerned about OS of patients with CRC and was a reliable and economic indicator to evaluate the prognosis of patients with CRC.

## 2 PATIENTS AND METHODS

### 2.1 Clinicopathological data and clinical characteristics

**FIGURE 1:**
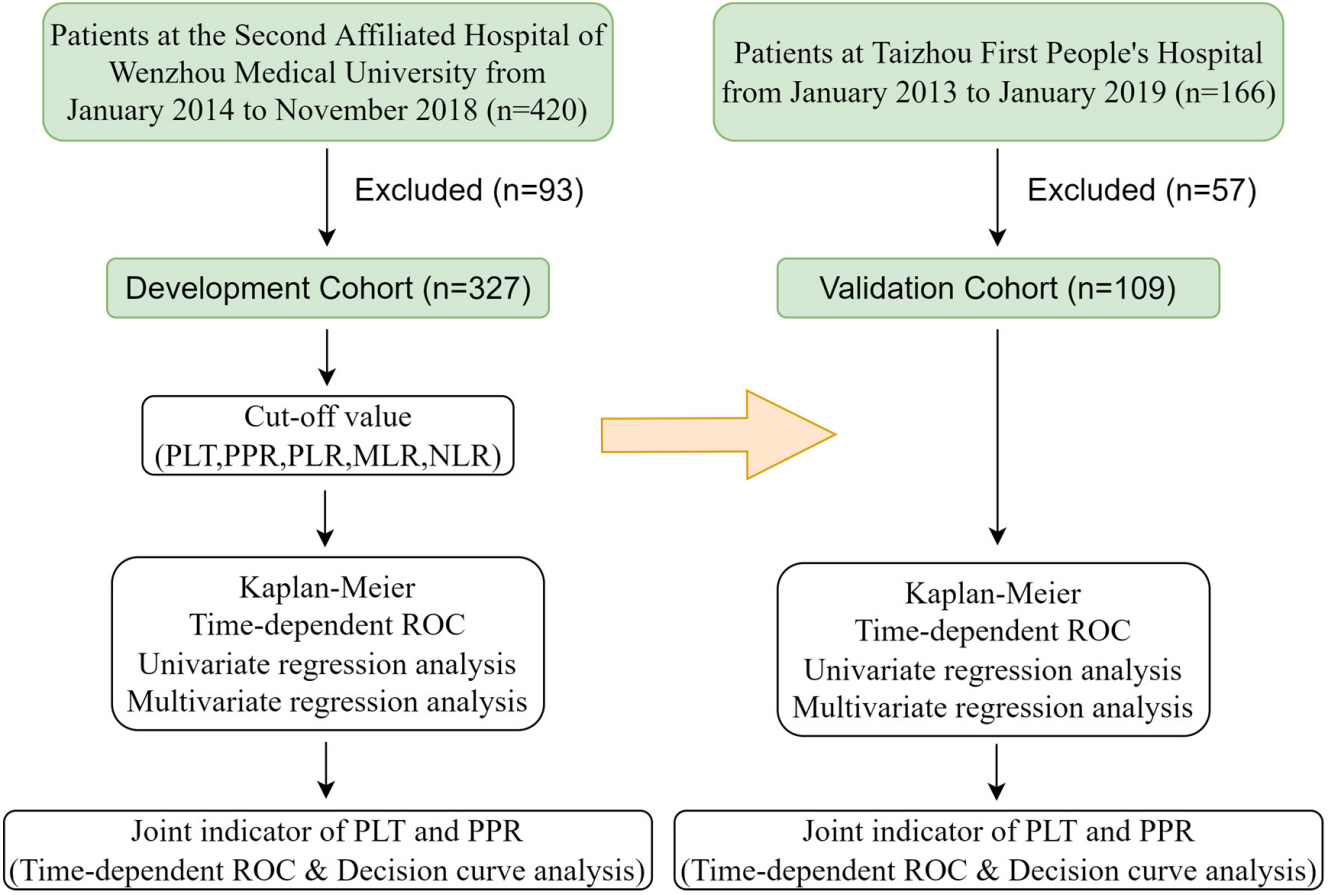
Flow chart of the criteria used to select the patients for inclusion in the present study. PPR, postoperative platelet/preoperative platelet ratio.

Two independent retrospective cohorts were used to train and validate the predictive model for the prognosis of CRC. As shown in Figure 1, patients with CRC (*n* = 420) who underwent radical resection of CRC at the Second Affiliated Hospital of Wenzhou Medical University from 2014 to 2018 were included as the development cohort. Considering the potential effect of neoadjuvant chemotherapy on myeloid cells, patients who received neoadjuvant chemotherapy before surgery were excluded from this cohort. Patients (*n* = 166) at Taizhou First People's Hospital from 2013 to 2019 were included as the validation cohort. All patients were confirmed by histopathological analysis. Patients meeting the following factors were excluded: (i) previous history of malignancies, (ii) history of antiplatelet therapy, (iii) accompanied by systemic lupus erythematosus, (iv) TNM judged as IV stages, (v) incomplete data, and (vi) lost to follow‐up. Eventually, 327 were enrolled eventually in the development cohorts and 109 patients in the validation cohorts, respectively. Clinical characteristics and clinicopathological data were recorded from the patients' medical records. These data included age, gender, TNM, tumor location, tumor size, histological grade, neurovascular invasion, distant metastasis, lymph node metastasis, and preoperative and postoperative peripheral blood count. The survival information of these patients was obtained through telephone calls and regular follow‐ups after the patients' operation. The TNM refers to American Joint Committee on Cancer, 8th. The time from operation to death or December 2020 was comprehended as the overall survival (OS), and the median follow‐up times were 44 months (range: 2–85 months) and 45 months (range: 3–96 months) in the development and validation cohorts, respectively.

### 2.2 Laboratory parameters

The survival time of platelets in the body is 5–7 days. In order to minimize the impact of surgical stress on platelet counts, platelet counts at least 2 weeks after surgery were used for the evaluation of PPR in the present study. Peripheral blood counts within 1 week before surgery were used for baseline values. In addition, considering the potential bone marrow transplantation effects of postoperative chemotherapy drugs, postoperative complete blood counts were collected before the first postoperative chemotherapy. The complete blood count from the development cohort was measured using the XE‐5000 hematology analyzer (Sysmex) and that from the validation cohort was detected by BC‐5390 hematology analyzer (Mindray). X‐tile software (ver.3.6.1) was devoted to determining the ideal cut‐off point.^25^ These values based on the development cohort were as follows: PLT, 230; PPR, 1.37; NLR, 3.66; PLR, 167.4; MLR, 0.24.

### 2.3 Statistical analysis

SPSS (v. 23.0, IBM, Inc.) and R (Version 3.6.3, R Core Development Team) were used for statistical analysis. The median (interquartile range) was devoted to the statistical description of nonnormally distributed continuous variables, and Mann–Whitney *U* test and Kruskal–Wallis test were used for differential analysis. Frequency and proportion were devoted to the statistical description of categorical variables, and χ^2^ test was devoted to statistical analysis. Kaplan–Meier analysis was performed for evaluating the OS. Time‐dependent receiver operating characteristic (ROC) curve was performed for evaluating the performance of predictive models. The association of risk factors with OS was determined by univariate and multivariable logistic regression analysis and variables with statistically significant differences in univariate survival analysis were further included in multivariate regression analysis. Logistic regression was used to build the joint indicator model. Decision curves were used to estimate the net benefit of the combined metrics of PLT and PPR. *p*‐value <0.05 was statistical significance.

## 3 RESULTS

### 3.1 Description of study cohorts

Clinical characteristics and clinicopathological data of patients were shown in Table 1. Important baseline variables showed a similar distribution in the two cohorts. These variables included age, gender, tumor location, TNM staging, tumor size, histological grade, and lymph node metastasis. Furthermore, male gender, rectum as the tumor location, G3–4 histological grade, stages II and III TNM staging, and tumor size <5 cm were higher frequency in the two cohorts. Significant differences in PLT and PPR were not found between the two groups. However, NLR, PLR, and MLR were significantly higher in the validation cohort than those in the development cohort. The survival curve for all patients were shown in Figure S1A. Importantly, significant difference was not found in OS probability between the two cohorts (Figure S1B). In addition, no significant statistical differences were found in OS between TNM stages I and II in the training group, and TNM stages I, II, and III in the validation group, although there was a trend toward differences between the groups (Figure S1C,D).

**TABLE 1:** Clinicopathological data and clinical characteristics of patients in the development and validation cohorts

Characteristics	Entire study (*n* = 436)	Development cohort (*n* = 327)	Validation cohort (*n* = 109)	*p* value
Age (years), median (range)	63 (34–91)	63 (34–91)	65 (41–88)	0.075
Gender, *n* (%)				0.646
Female	159 (36%)	122 (37%)	38 (35%)	
Male	277 (64%)	205 (63%)	71 (65%)	
Tumor location, *n* (%)				1.000
Colon	168 (39%)	126 (39%)	42 (39%)	
Rectum	268 (61%)	201 (61%)	67 (61%)	
Histological grade, *n* (%)				0.236
G1‐2	51 (12%)	38 (12%)	13 (12%)	
G3‐4	353 (81%)	261 (80%)	92 (84%)	
No record	32 (7%)	28 (8%)	4 (4%)	
pT category, *n* (%)				0.054
pT1‐2	97 (22%)	80 (24%)	17 (16%)	
pT3‐4	339 (78%)	247 (76%)	92 (84%)	
pN category, *n* (%)				0.091
pN X/0	258 (59%)	201 (61%)	57 (52%)	
pN+	178 (41%)	126 (39%)	52 (48%)	
TNM stage (AJCC, 8th) *n* (%)				0.050
I	85 (19%)	72 (22%)	13 (12%)	
II	173 (40%)	129 (39%)	44 (40%)	
III	178 (41%)	126 (39%)	52 (48%)	
Tumor size (cm), *n* (%)				0.233
<5	300 (69%)	220 (67%)	80 (73%)	
≥5	136 (31%)	107 (33%)	29 (27%)	
Neurovascular invasion, *n* (%)				<0.001
No	300 (69%)	245 (75%)	55 (50%)	
Yes	136 (31%)	82 (25%)	54 (50%)	
PLT (×10^9^/L), median (IQR)	238 (198–290)	238 (201–299)	241 (190–282)	0.301
NLR, median (IQR)	2.17 (1.66–3.30)	2.12 (1.64–3.05)	2.90 (1.70–5.45)	0.001
PLR, median (IQR)	142 (109–196)	139 (105–187)	159 (115–217)	0.021
MLR, median (IQR)	0.22 (0.17–0.32)	0.21 (0.17–0.31)	0.25 (0.18–0.40)	0.048
PPR, median (IQR)	1.08 (0.88–1.34)	1.08 (0.89–1.34)	1.08 (0.87–1.35)	0.812

Abbreviations: AJCC, American Joint Committee on Cancer; MLR, monocyte/lymphocyte ratio; NLR; neutrophil/lymphocyte ratio; PLR, platelet/lymphocyte ratio; PLT, platelet count; PPR, Postoperative platelet/preoperative platelet ratio.

### 3.2 Survival analysis

**FIGURE 2:**
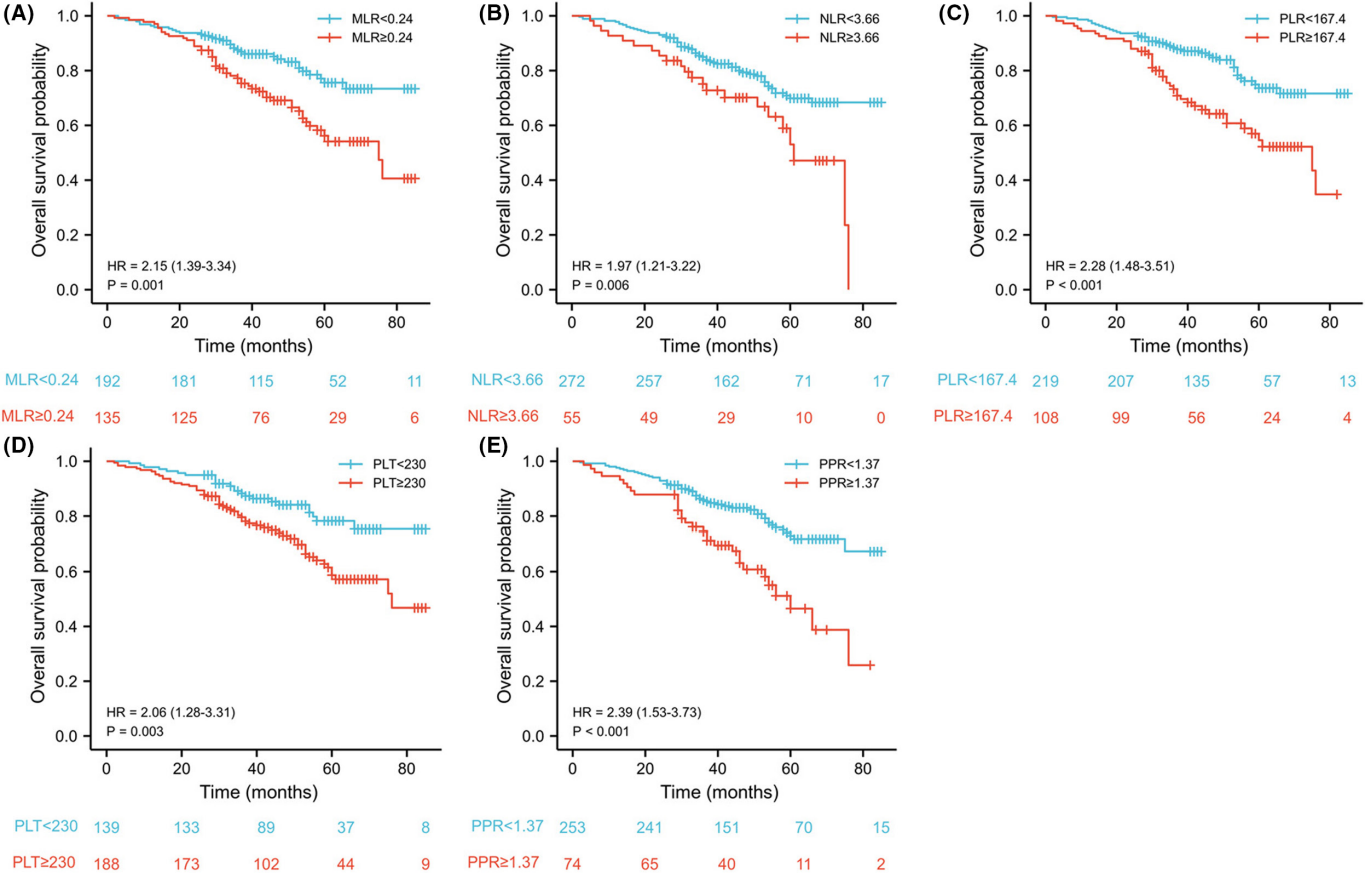
The prognostic significance of the monocyte/lymphocyte ratio (MLR) (A), neutrophil/lymphocyte ratio (NLR) (B), platelet/lymphocyte ratio (PLR) (C), platelet count (PLT) (D), and postoperative platelet/preoperative platelet ratio (PPR) (E) in patients with colorectal cancer in the development cohort. PLT, platelet count; NLR; neutrophil/lymphocyte ratio; PLR, platelet/lymphocyte ratio; MLR, monocyte/lymphocyte ratio; PPR, Postoperative platelet/preoperative platelet ratio.

**FIGURE 3:**
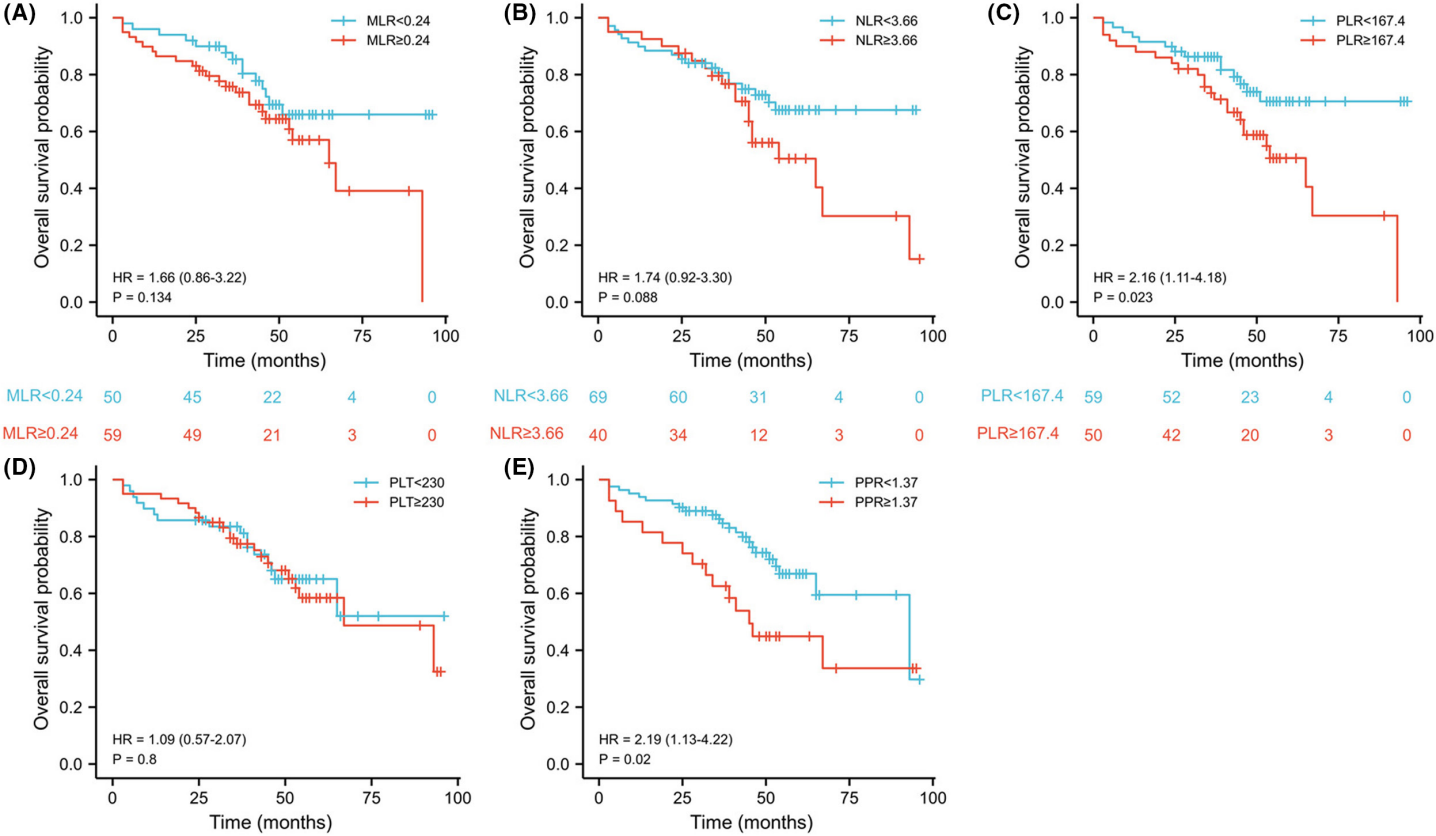
The prognostic significance of the monocyte/lymphocyte ratio (MLR) (A), neutrophil/lymphocyte ratio (NLR) (B), platelet/lymphocyte ratio (PLR) (C), platelet count (PLT) (D), and postoperative platelet/preoperative platelet ratio (PPR) (E) in patients with colorectal cancer in the validation cohort. PLT, platelet count; NLR; neutrophil/lymphocyte ratio; PLR, platelet/lymphocyte ratio; MLR, monocyte/lymphocyte ratio; PPR, Postoperative platelet/preoperative platelet ratio.

The correlation between prognosis and PLT, NLR, PLR, MLR, or PPR was further analyzed. In the development cohort, high PLT (≥230 × 10^9^/L), NLR (≥3.66), PLR (≥167.4), MLR (≥0.24), and PPR (≥1.37) were significantly in connection with the poor OS of patients undergoing radical resection of CRC (hazard ratio [HR]:2.06 [1.28–3.31], *p* = 0.003; HR: 1.97 [1.21–3.22], *p* = 0.006; HR:2.28 [1.48–3.51], *p* < 0.001; HR:2.15 [1.39–3.34]; HR: 2.39 [1.53–3.73], *p* < 0.001; respectively) (Figure 2). In validation cohort, high PLR and PPR were significantly in connection with the poor OS of patients undergoing radical resection of CRC (HR:2.16 [1.11–4.18], *p* = 0.023 and HR: 2.19 [1.13–4.22], *p* = 0.020), but the NLR, PLR, and PLT were the opposite (Figure 3).

### 3.3 Univariate and multivariate analyses

Univariate and multivariate regression analyses were further performed on risk indicators and baseline characteristics. In development cohort, univariate analysis showed that TNM stage, postoperative chemotherapy, PPR, PLT, NLR, PLR, and MLR were remarkable prognostic factors for patients undergoing radical resection of CRC, and multivariate analysis found that TNM stage, PLT, and PPR were independent predictor (Table 2). In the validation cohort, univariate analysis showed that postoperative chemotherapy, NLR, PLR, and PPR were remarkable prognostic factors, and multivariate analysis showed that PLR and PPR were independent predictors (Table 3). The above indicated that PPR was an independent predictor for the OS of patients with CRC and showed a better value as a prognostic indicator than PLT, NLR, PLR, and PPR.

**TABLE 2:** Univariate and multivariate cox regression analyses for overall survival in patients with colorectal cancer of development cohort

Characteristics	Univariate analysis		Multivariate analysis	
	Hazard ratio (95% CI)	*p* value	Hazard ratio (95% CI)	*p* value
Sex				
Male vs Female	0.77 (0.49–1.19)	0.241		
TNM stage				
Stage II vs Stage I	1.82 (0.86–3.87)	0.121	1.65 (0.77–3.55)	0.200
Stage III vs Stage I	3.65 (1.79–7.46)	<0.001	4.06 (1.97–8.37)	<0.001
Location				
Colon vs Rectum	0.93 (0.59–1.45)	0.742		
Size				
<5 cm vs ≥5 cm	1.24 (0.79–1.93)	0.345		
Neurovascular invasion				
No vs Yes	0.91 (0.54–1.55)	0.738		
Chemotherapy				
No vs Yes	1.97 (1.23–3.17)	0.005	1.47 (0.86–2.53)	0.163
PLT				
<230 vs ≥230	2.06 (1.28–3.31)	0.003	2.12 (1.24–3.65)	0.006
NLR				
<3.66 vs ≥3.66	1.97 (1.21–3.22)	0.006	1.00 (0.55–1.83)	0.989
PLR				
<167.4 vs ≥167.4	2.28 (1.49–3.51)	<0.001	1.59 (0.88–2.86)	0.125
MLR				
<0.24 vs ≥0.24	2.15 (1.39–3.34)	<0.001	1.61 (0.94–2.76)	0.084
PPR				
<1.37 vs ≥1.37	2.39 (1.53–3.73)	<0.001	2.64 (1.67–4.18)	<0.001

Abbreviations: CI, Confidence interval; MLR, monocyte/lymphocyte ratio; NLR; neutrophil/lymphocyte ratio; PLR, platelet/lymphocyte ratio; PLT, platelet count; PPR, Postoperative platelet/preoperative platelet ratio.

**TABLE 3:** Univariate and multivariate cox regression analyses for overall survival in patients with colorectal cancer of validation cohort

Characteristics	Univariate analysis		Multivariate analysis	
	Hazard ratio (95% CI)	*p* value	Hazard ratio (95% CI)	*p* value
Sex				
Male vs Female	1.10 (0.56–2.15)	0.783		
TNM stage				
Stage II vs Stage I	2.99 (0.68–13.13)	0.146		
Stage III vs Stage I	3.40 (0.80–14.52)	0.099		
Location				
Colon vs Rectum	0.79 (0.41–1.49)	0.460		
Size				
<5 cm vs ≥5 cm	0.90 (0.44–1.86)	0.778		
Neurovascular invasion				
No vs Yes	1.20 (0.63–2.28)	0.572		
Chemotherapy				
No vs Yes	2.38 (1.18–4.82)	0.016	1.84 (0.89–3.78)	0.099
PLT				
<230 vs ≥230	1.09 (0.57–2.07)	0.800		
NLR				
<3.66 vs ≥3.66	1.74 (0.92–3.30)	0.088	1.22 (0.57–2.58)	0.612
PLR				
<167.4 vs ≥167.4	2.16 (1.11–4.18)	0.023	2.33 (1.05–5.18)	0.038
MLR				
<0.24 vs ≥0.24	1.66 (0.86–3.22)	0.134		
PPR				
<1.37 vs ≥1.37	2.19 (1.13–4.22)	0.020	2.67 (1.37–5.23)	0.004

Abbreviations: PLT, platelet count; NLR; neutrophil/lymphocyte ratio; PLR, platelet/lymphocyte ratio; MLR, monocyte/lymphocyte ratio; PPR, Postoperative platelet/preoperative platelet ratio; CI, Confidence interval.

### 3.4 Predictive value for prognosis of various parameters

**FIGURE 4:**
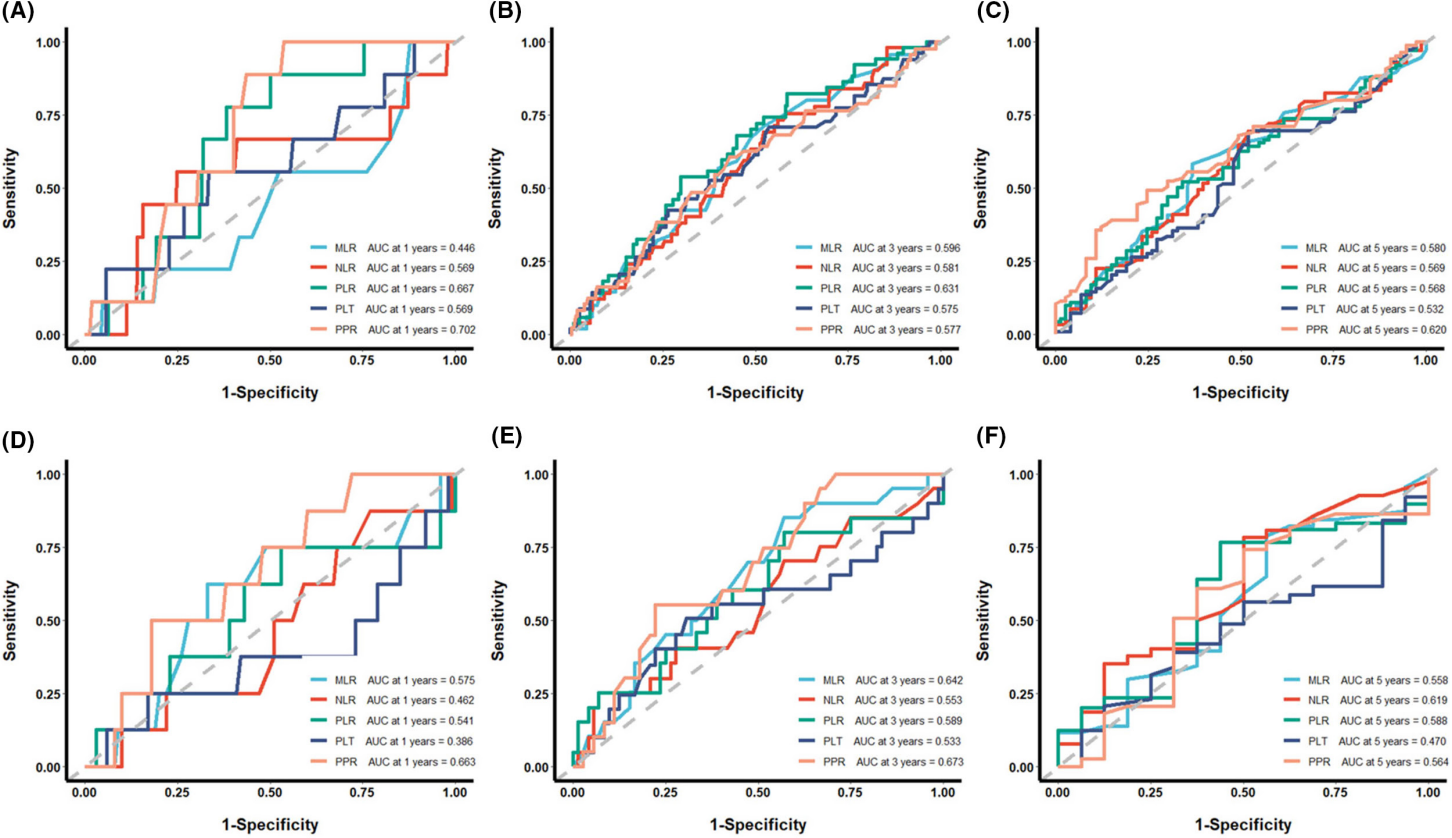
The predictive ability of the MLR, NLR, PLR, PLT, and PPR in colorectal cancer by time‐dependent receiver operating characteristic (ROC) curves in 1 year, 3 years, and 5 years in the development cohort (A–C) and validation cohort (D–F). PLT, platelet count; NLR; neutrophil/lymphocyte ratio; PLR, platelet/lymphocyte ratio; MLR, monocyte/lymphocyte ratio; PPR, Postoperative platelet/preoperative platelet ratio.

According to the time‐dependent ROC curve, the areas under the curves (AUCs) of PPR, namely, 0.702 and 0.620 for 1‐year and 5‐year OS of patients undergoing radical resection of CRC, respectively, were higher than those of MLR, PLR, PLT, and NLR in the development cohort (Figure 4A,C). The AUC of PPR (0.577) for the 3‐year OS was the third largest area among the five indexes (Figure 4B). In the validation cohort, the AUC for the 1‐year and 3‐year OS of PPR of such patients were higher than those of MLR, PLR, PLT, and NLR (Figure 4D,E). The AUC of PPR (0.564) for the 5‐year OS was the third largest in the five indexes (Figure 4F).

### 3.5 Prognostic value of joint index

**FIGURE 5:**
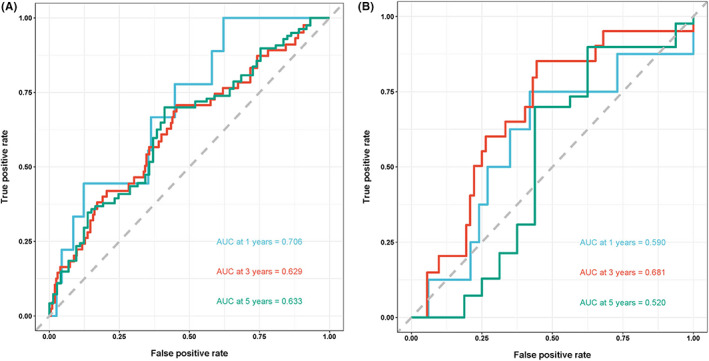
The predictive ability of the joint index of platelet count (PLT) and postoperative platelet/preoperative platelet ratio (PPR) in colorectal cancer by time‐dependent receiver operating characteristic (ROC) curves in 1 year, 3 years, and 5 years in the development cohort (A) and validation cohort (B).

The effect of combining PPR and PLT in the prognosis of CRC was further evaluated due to the good performance of PLT in the development cohort. The joint parameter showed higher AUCs for the 1‐year and 5‐year OS in the development cohort and 3‐year OS in the validation cohort than single index. In addition, the joint parameter showed second‐best AUC for the 3‐year OS in the development cohort and 1‐year OS in the validation cohort compared with the single index (Figure 5). Furthermore, the decision curve shows that in the development cohort, the net benefit of joint index in predicting the prognosis of CRC is the highest, followed by PPR and PLT. In the validation cohort, the net benefit of PPR is the highest, followed by joint index and PLT (Figure S2). These results suggested that PPR and the joint index of PLT and PPR were a trustworthy indicator for assessing the prognosis.

## 4 DISCUSSION

Over the last decades, an abundant amount of research has focused on the mechanism of platelet involvement in tumor growth and metastasis. Activated platelets mediated by tumor cells could extravasate into the tumor microenvironment and release cytokines and tumor growth factors to promote epithelial‐mesenchymal transition^26^, ^27^ and induce drug resistance of cancer cells.^28^ The interplay of galectin 3 of tumor cells and platelet glycoprotein VI (GPVI) promotes tumor metastasis,^29^ and Hsp47 enhances the interplay between tumor cells and platelets and further promotes tumor metastasis.^30^ Zhou et al.^31^ and Xu et al.^32^ testified that blocking the interplay of platelet and tumor cell prevents tumor metastasis and reverses tumor immunosuppression.

The usefulness of preoperative platelet count for prognostic assessment in patients with solid tumors has been extensively studied. However, several issues have to be studied. First, preoperative platelet counts cannot be standardized and individualized for clinical use due to the variability among individuals, instruments, and regions. Second, the relevance of increased postoperative platelet count and tumor resurgence and poor prognosis remains unknown. Here, the PPR values were calculated based on the post‐ and pre‐operative platelet counts and thus could, at least to some extent, normalize the differences in the baseline values due to individual, regional and instrument variations. Hence, PPR can better reflect the individual's platelet dynamics and may be considered a standardized indicator of individual platelets. Hence, PPR, a standardized indicator of platelet count, was used to assess the relevance of postoperative platelet counts to the survival outcome of patients undergoing radical resection of CRC.

In the present study, the usefulness of MLR, NLR, preoperative PLT, PPR and PLR for prognostic assessment in patients with CRC was evaluated in two independent centers. Elevated PPR was enormously relevant to poor prognosis in patients undergoing radical resection of CRC and was an independent predictor for the OS in the development and validation cohorts. Importantly, PPR showed a pleasing value in foreseeing the prognosis of such patients when compared with PLT, NLR, PLR, and MLR in the two cohorts. These results indicated that persistent elevation of postoperative platelets was associated with poor patient outcomes and that increased platelets might take a favorable role in the resurgence and proliferation of tumor cells after surgery. Dynamic assessment of postoperative platelet might be indicative of patient prognosis.

Elevated preoperative PLT was also significantly relevant to poor prognosis and was an independent predictor in the development cohort, which was to be identical to previous research results.^8^, ^10^, ^11^ However, it was not relevant to prognosis in the validation cohort, which might be due to differences in the hematology analyzer and regions of the two centers. In addition, MLR, NLR, and PLR were markedly relevant to the short survival time of the patients, but they were not independent predictors of OS of patients in the development cohort. Therefore, only PLT and PPR were included in the further analyses of the combined indicators to predict the prognostic value for patients undergoing radical resection of CRC. The combined metrics of PLT and PPR interestingly showed better predictive value compared with the single indicator of PPR in the two cohorts. Furthermore, PLT, PPR, and the joint index appeared better predictive usefulness in 1‐year and 3‐year OS compared to 5‐year OS, possibly due to the limited number of patients with 5‐year OS data. Because only patients included in 2013, 2014, and 2015 year had 5‐year survival data, while more patients had 1‐year and 3‐year OS data. Notably, we found that postoperative chemotherapy was significantly associated with patient prognosis, but it was not an independent predictor of patient prognosis. The reason might be that the proportion of patients with different TNM stages undergoing postoperative chemotherapy was different. Although very few patients with stage I underwent postoperative chemotherapy, they might show a better prognosis than patients with advanced‐stage who receive chemotherapy.

The peripheral blood count is an essential item in physical examination before surgery and follow‐up after surgery. Accordingly, PPR is easily available and economical and has good repeatability. However, this study has several restrictions. (i) Preoperative and postoperative peripheral blood counts need to be performed at the same institution. (ii) The queue included in the present analysis was a retrospective cohort, and further prospective, multi‐center, and large‐sample studies are needed for optimal cut‐off value of PPR and the usefulness of prognostic assessment of PPR. (iii) Progression‐free survival was not incorporated into the analysis due to the follow‐up being different in different hospitals and the follow‐up being sometimes irregular in the dual‐center.

## 5 CONCLUSIONS

PPR was a trustworthy index to assess the survival outcome of patients undergoing radical resection of CRC. Elevated PPR was closely related to poor prognosis of such patients. As a noninvasive, economical, and easily available indicator, PPR was an objective and standardized predictor of the survival of patients undergoing radical resection of CRC. And it would also give clinicians a hand with stratifying the mortality risk of such patients and draught suitable individualized treatment plans.

## AUTHOR CONTRIBUTIONS

Chunming Ding and Minghua Jiang designed the project. Wei Yang, Xiaoying Zheng, Minghui Wu, Fengming Zhang, Shuizhi Xu, Xiuchao Wang, Menghui Song, Chang You, and Ting Zhang acquired the data. Wei Yang, Xiaoying Zheng, and Minghui Wu analyzed and interpreted the data. Fengming Zhang, Shuizhi Xu, Xiuchao Wang, Menghui Song, Chang You, and Ting Zhang inspected the results. Wei Yang, and Xiaoying Zheng drafted the manuscript. Chunming Ding, Minghua Jiang, Wei Yang, Xiaoying Zheng, Minghui Wu, Fengming Zhang, Shuizhi Xu, Xiuchao Wang, Menghui Song, Chang You, and Ting Zhang participated in the proofreading.

## FUNDING INFORMATION

This study was supported by the High‐Level Innovation Team of Universities in Zhejiang Province (Grant 604090352/610)and the Innovation Discipline of Zhejiang Province in Nucleic Acid Molecular Diagnostics (Grant 437201702G).

## CONFLICT OF INTEREST

No conflicts of interest.

## ETHICS APPROVAL

The patient's data came from the hospital's big data platform, which does not require the patient's informed consent because it is a retrospective study.

## Supporting information


Figure S1

Figure S2

## Data Availability

The data used in this study are available on putting in for the relevant authors on reasonable demand.
