# Supplementary material for: Development and validation of postoperative and preoperative platelets ratio (PPR) to predict the prognosis of patients undergoing surgery for colorectal cancer: A dual‐center retrospective cohort study
Source: Cancer Med. 2022 Jun 11;12(1):111–21. doi: 10.1002/cam4.4930 (PMC9844599; doi:10.1002/cam4.4930)
Supplement: Supplementary file 1 — Figure S1 [file CAM4-12-111-s001.pdf]

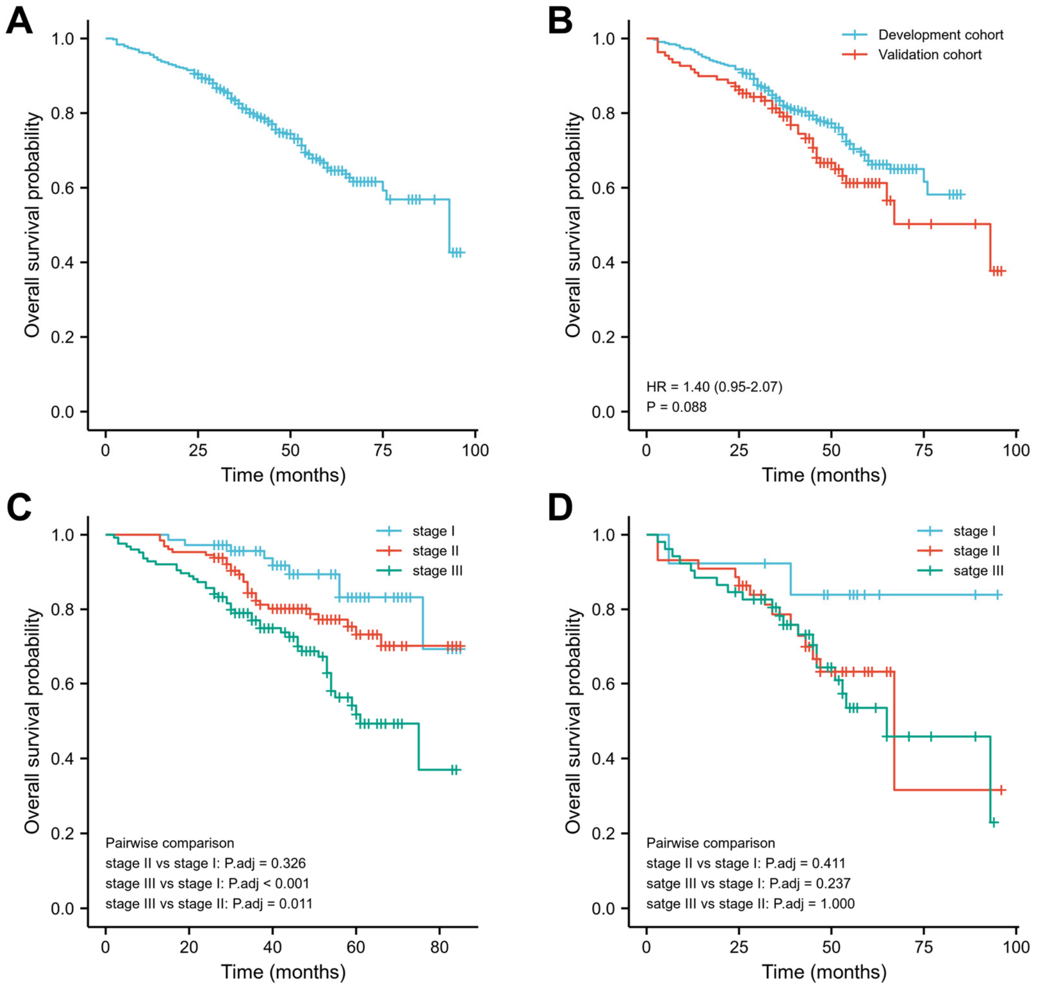

Figure S1 Prognostic information of included patients. Survival curve for all patients (A) and patients in the development cohort and validation cohort (B). Survival curve for patients of development cohort (C) and validation cohort (D) in different TNM staging.
