# Supplementary material for: Development and validation of postoperative and preoperative platelets ratio (PPR) to predict the prognosis of patients undergoing surgery for colorectal cancer: A dual‐center retrospective cohort study
Source: Cancer Med. 2022 Jun 11;12(1):111–21. doi: 10.1002/cam4.4930 (PMC9844599; doi:10.1002/cam4.4930)
Supplement: Supplementary file 2 — Figure S2 [file CAM4-12-111-s002.pdf]

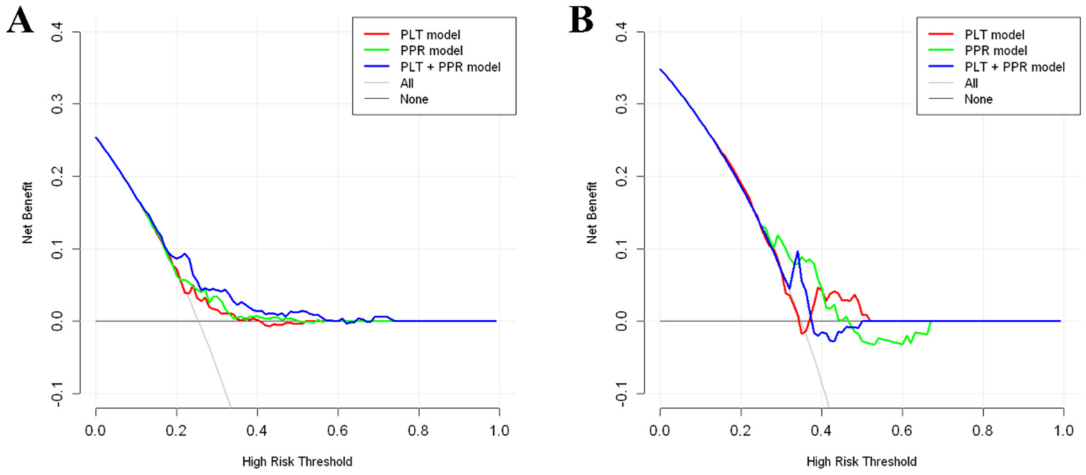

Figure S2 Decision curve analysis of PLT, PPR, and joint index in development cohort (A) and validation cohort (B).
